# Time for You: A process evaluation of the rapid implementation of a multi-level mental health support intervention for frontline workers during the COVID-19 pandemic

**DOI:** 10.1371/journal.pone.0293393

**Published:** 2023-10-27

**Authors:** Bryan McCann, Simon C. Hunter, Kareena McAloney-Kocaman, Paul McCarthy, Jan Smith, Eileen Calveley

**Affiliations:** 1 Department of Psychology, School of Health and Life Sciences, Glasgow Caledonian University, Glasgow, United Kingdom; 2 Graduate School of Education, University of Western Australia, Perth, Western Australia, Australia; The University of Alabama, UNITED STATES

## Abstract

The coronavirus (COVID-19) pandemic had wide-ranging negative impacts on mental health. The pandemic also placed extraordinary strain on frontline workers who were required to continue working and putting themselves at risk to provide essential services at a time when their normal support mechanisms may not have been available. This paper presents an evaluation of the Time for You service, a rapidly developed and implemented intervention aimed at providing frontline workers with quick access to flexible online mental health support. Time for You provided service users with three service options: self-guided online cognitive behavioural therapy (CBT) resources; guided engagement with online CBT resources; 1–1 psychological therapy with trainee sport and exercise psychologists and trainee health psychologists. A process evaluation informed by the Consolidated Framework for Implementation Research considered service fidelity, adaptations, perceived impact, reach, barriers, and facilitators. Interviews with project managers (n = 5), delivery staff (n = 10), and service users (n = 14) explored perceptions of the service implementation and outcomes, supported by data regarding engagement with the online CBT platform (n = 217). Findings indicated that service users valued the flexibility of the service and the speed with which they were able to access support. The support offered by Trainee Psychologists was perceived to be of high quality, and the service was perceived by service users to have improved mental health and wellbeing. The rapid implementation contributed to issues regarding appropriate service user screening that led to trainee psychologists being unable to provide the service users with the support they needed as the presenting issues were outside of trainees’ competencies. Overall, the findings suggest that interventions offering flexible, online psychological support to frontline workers can be an effective model for future interventions. Trainee psychologists are also able to play an important role in delivering such services when clear screening processes are in place.

## Introduction

The mental health impacts of the COVID-19 pandemic are wide-ranging [1–3], and adults exhibited vulnerability at different periods throughout this public health emergency [4, 5]. The pandemic represented an unprecedented pressure on key workers such as health and social care, transport and retail staff, placing them on the frontline of servicing the nation’s essential needs while being exposed to COVID-19 itself [6, 7]. Reduced participation in social activities resulting from pandemic-related lockdown measures is also likely to have been detrimental to psychological wellbeing [8]. At this critical juncture, the curtailment of existing channels of support, such as General Practitioners and mental health services, compounded the difficulties that were experienced by many workers [9]. While some workplace support schemes were available, barriers to the uptake of these included heavy workload, understaffing, inconvenient locations, and the stigma of being judged [9].

The primary mental health difficulties associated with the COVID-19 pandemic are symptoms of depression and anxiety. Such symptoms indicating mental health difficulties have been reported across a broad cross-section of society including pregnant women [10], adults with disabilities [11], teachers [12], high school and University students [13, 14], and patients with rare diseases [15]. These findings are seen across the globe, including Canada [16], the United Kingdom [UK; 17], China [18], Singapore [19], Turkey [20] the United States of America (USA) [21], and elsewhere [22]. Particularly relevant to the current study are reports of increased symptomatology among healthcare workers [e.g., 19, 23–28] including those working within the Scottish context [29]. The experience of mental health difficulties among healthcare workers is particularly concerning at a time when society most needs their support and when healthcare workers are reluctant to seek professional help [28, 29]. There is therefore a strong argument to make interventions available that are suitable to the healthcare worker population in the context of the pandemic [30].

The present study reports an evaluation of a mental health intervention, Time for You, delivered online, focussing specifically on frontline workers who perceived themselves as having poorer mental health during the COVID-19 pandemic. This service offered frontline workers with immediate access to online mental health support via three routes: online, self-guided internet-delivered cognitive behaviour therapy (iCBT); supported completion of iCBT resources; and 1–1 psychological therapy delivered by trainee psychologists.

The online *Living Life to the Full* (LLTTF) support platform was used within the Time for You intervention and offers an evidence-based iCBT approach that is appropriate for tackling symptoms of both depression and anxiety [31–33]. National Institute for Health and Care Excellence (NICE) [34] guidelines support the use of psychological therapies for managing depression and anxiety disorders, advocating a stepped care approach depending on the intensity of risk and symptoms. The guidelines consider low intensity approaches to be appropriate for mild-to-moderate depression, which include computer-delivered cognitive behaviour therapy (cCBT) or iCBT delivered with 6–8 short support sessions over a 10–12 week period. Recent reviews and meta-analyses have established iCBT as an effective and acceptable approach for managing anxiety and depressive disorders, showing significantly improved outcomes in comparison with control groups on a par with face-to-face CBT therapy [35, 36].

Trainee Sport and Exercise Psychologists and trainee Health Psychologists provided online 1–1 psychological support to frontline workers as part of the Time for You service evaluated in this paper as they had received the necessary significant training and supervised experience in person-centred and cognitive-behavioural therapy as part of the programmes delivered by the academic partner. NICE [34] suggests that longer counselling-based support sessions may be of benefit when low intensity treatments have proven less effective. However, one barrier to such provision is the existing workload of mental healthcare professionals, with lengthy waiting lists that are already a problem in the UK [37] and which are likely to only get worse [38]. In this context, trainee psychologists offer a potentially rich seam of support, with trainee counselling psychologists’ work being found to be as effective in improving client psychological outcomes as experienced therapists when under close supervision [39]. However, there is less knowledge about outcomes for trainees in disciplines other than clinical or counselling psychology.

The Medical Research Council guidance on evaluating complex interventions [40] offers a framework within which to evaluate the implementation and effectiveness of an iCBT service which is here supported by trainee psychologists offering longer counselling-based support sessions. The process evaluation reported herein, therefore focused on: adherence to the original plan, along with adaptations; the reach of the intervention; and barriers and facilitators relating to the implementation context. This approach to evaluation was complimented by the use of the Consolidated Framework for Implementation Research (CFIR) which was used to guide a systematic analysis of barriers and facilitators to implementation [41, 42]. Specific evaluation objectives were to:

1. Explore facilitators and barriers to implementation of Time for You and variations from original plans.
2. Explore implementation contexts (including intervention uptake; demographics; and engagement with service).
3. Explore the acceptability and impact of Time for You amongst participating frontline workers.

## Materials and methods

### The Time for You intervention

The Scottish Association for Mental Health (SAMH) is a Scottish charity which focusses on mental health support and information. SAMH was acutely aware of the pressures placed on frontline workers through reports from its own social care services as well as those of partner organisations [43]. SAMH had experience of integrating the LLTTF platform and wellbeing coaching with previous services, and had identified through stakeholder engagement a need to develop capacity in psychological support (e.g., therapy), all of which were factors informing the development of a new service for frontline workers. In partnership with Glasgow Caledonian University (GCU), SAMH rapidly developed over the period of a month in the summer of 2020 a flexible and accessible intervention that could offer different levels of mental health support to meet the immediate needs of a working population, using evidence-based approaches and informed by SAMH’s previous services. This intervention, called the *Time for You* service, was designed by SAMH’s Psychological Wellbeing team and GCU’s Department of Psychology staff to support frontline workers with mild-to-moderate mental health and wellbeing challenges by providing immediate access to three online mental health support options. When funding for the service was approved in September 2020, rapid implementation began in December 2020, managed by SAMH, and Time for You was formally launched in April 2021, coinciding with the start of the third wave of the COVID-19 pandemic in the UK. The service was promoted via SAMH and GCU’s social media channels and press releases, and via partner organisations such as local authority organisations and regional National Health Service (NHS) Boards. The service was initially designed to offer three support options as described below.

#### Option 1

Frontline workers gained access to the LLTTF online software. LLTTF is an online platform of resources supporting individuals in improving and managing their mental health, which was adapted to acknowledge the impact of COVID-19 and associated lockdowns. Cognitive behaviour therapy (CBT) theory and principles inform this platform. Frontline workers could register and work through the various resources and tasks in a self-guided and self-paced manner, and/or request support via one of the other two options for the service.

#### Option 2

A SAMH Wellbeing Practitioner supported frontline workers in the completion of the LLTTF resources. The Wellbeing Practitioners received training in LLTTF resources and coached frontline workers work through LLTTF tasks for an expected 20–30 minutes of support per week.

#### Option 3

Frontline workers accessed a series of 1–1 psychological support sessions with sport and exercise or health trainee psychologists enrolled on the doctorate in psychology programme at GCU. These trainees had gained training and experience in supporting service users through person-centred therapy and CBT modalities during their studies. To support their work with clients, trainees also had regular individual supervision with a qualified practitioner (1hr of supervision for every 8hrs of client work), participated in weekly group supervision sessions as part of their studies, and took part in fortnightly group reflective sessions with other trainees delivering the service and facilitated by Time for You delivery managers. The resulting Time for You intervention used an adapted version of Five Areas Ltd LLTTF online support platform as a basis for and access to the service. Frontline workers were given automatic access to Option 1 when registering for the service but where they requested Options 2 or 3 they were triaged to determine their individual needs and appropriateness for the service. Triaging included potential service users completing the Warwick-Edinburgh Mental Well-being Scale (WEMWBS) [44] via the LLTTF platform. The WEMWBS results were used alongside an in-depth assessment discussion between the frontline worker and the Time for You service manager to determine which service option best suited the frontline worker’s needs and that these needs were in line with the service aims to provide support with mild-to-moderate mental health challenges.

### Participants

All those involved in developing, implementing, and delivering the Time for You service were invited to take part in interviews to share their experience and perceptions of its implementation and impact. In total, five service developers/managers; five wellbeing practitioners/option 2 managers; and five trainee psychologists participated, with one trainee psychologist being interviewed at two time points.

All service users were invited to give consent to allow data gathered on the LLTTF platform to be shared with the evaluation team. Recruitment for the interviews began in August 2021 to allow the service to manage any early issues. From 132 invitations, 16 service users agreed to take part in interviews, 14 of whom participated. Five of the service users interviewed had gained support via Option 1 (self-directed LLTTF access), two service users interviewed had gained support via Option 2 (SAMH Wellbeing Practitioner facilitated support), and seven service users were interviewed who had gained support via Option 3 (psychological support from trainee psychologist). The aim had been to interview at least ten percent of the number of service users receiving support from each service option given research resource limitations, which was achieved for Option 2 and 3, but not for Option 1.

### Measures

The evaluation explored the service implementation processes, barriers and facilitators to effective implementation, implementation contexts, and the acceptability of Time for You. Semi-structured interview guides (S1 File) were used that aimed to provide answers to the specific research questions, while allowing a degree of flexibility to explore emergent perceptions and experiences.

Service developers and managers answered questions in relation to the service design process, including the perceived need that prompted, and approaches that informed its development. Questions also explored how closely the implemented service related to initial service plans, and the causes for any deviations from those plans.

Service delivery staff, including Option 2 managers and trainee psychologists, answered questions about the nature of the support they provided to frontline workers, including any changes to the planned support and the reasons for changes. Questions also related to the engagement of frontline workers in Options 2 and 3 of the service, and what delivery staff felt were the outcomes for the frontline workers they worked with.

Frontline workers answered questions relating to the nature and experience of their work during the COVID-19 pandemic. Questions also explored the reasons for which frontline workers had sought support from the Time for You service, and their experience of the service option from which they had been gaining support. Frontline workers also answered questions about the extent to which the support they had received had helped them address the reasons they sought support in the first place, and how the Time for You service might be improved.

### Procedure

After gaining ethical approval from the Psychology, Social Work, and Allied Health Sciences Ethics Committee at GCU, all Time for You development and delivery staff, including trainee psychologists, were sent invitations to participate in interviews which included an information sheet and consent. The LLTTF landing page for the service provided a brief information sheet and a consent form for service users inviting them to participate in interviews. Semi-structured interviews took place via either telephone or Microsoft Teams and lasted approximately 30–60 minutes. All participants verbally provided oral consent before commencing the interviews.

### Analysis

Transcription of interviews followed an intelligent verbatim approach. Transcripts were anonymised and entered into QSR NVivo (v12) retaining the study ID number and including codes for service option and participants’ role in the evaluation. Framework analysis complemented by inductive thematic analysis was used to summarise issues raised by participants about the implementation of Time for You, its acceptability, and its effectiveness in addressing wellbeing and suggestions for improvement. Analysis focused on identifying how the intervention worked, for whom, and the impact of the tiered support approach. The Principal Investigator double coded a proportion of transcripts and ongoing discussions amongst the research team took place regularly during the analysis programme regarding identified themes. The process generated a codebook using all 39 CFIR constructs to ensure any contextual factors which may have influenced implementation were represented [41]. Data were coded in relation to key analytic themes (CFIR factor), with sub-codes used to identify factors peculiar to the Time for You intervention. Concurrent inductive thematic analysis was carried out to capture any additional factors relating to implementation and particularly, to explore impact on service users, following the steps outlined by Clarke et al. [45]. Researchers ensured maximum representation of ideas by labelling some text with multiple tags or codes.

## Results

### Intervention reach and user demographics

Of the 290 service users who registered, 217 consented to share data and take part in the evaluation. Ninety service users self-referred to options 2 or 3. Following assessment where possible, 24 service users were referred to Option 2 and 40 to Option 3. Table 1 shows the number of registrations by option each month during the evaluation period. Fourteen cases were closed due to either being inappropriate for the service (5), not responding to contact (7), or preferring to access other support (2). By the end of November 2021, 11 Option 2 service users had consented to share data and 26 Option 3 service users had given this consent.

**Table 1:** Number of registrations for the Time for You service options per month in 2021.

	Apr	May	Jun	Jul	Aug	Sept	Oct	Nov
**Option 1**	27	49	64	32	20	10	19	12
**Option 2**	0	1	3	0	6	4	6	4
**Option 3**	2	1	3	2	7	7	7	11

The average age of those registering on the service was 38.5 years (SD = 12.2; range 18 to 65 years). Table 2 shows the number of service users within each age category. Frontline workers from 31 out of the 32 local authority areas in Scotland accessed the Time for You service, with Shetland being the only local authority area not represented within the sample. Service users were mainly based in the following locations: City of Glasgow (80), North Lanarkshire and South Lanarkshire (55) and Aberdeen City/ Aberdeenshire (26). From the eight employment sectors in the full data set, the highest number of service users were in the Health Care sector (106) followed by Social Care (94) and Education (37). Table 3 shows the variety of employment sectors represented within the sample and the number of services users within each sector.

**Table 2:** Number of service users in each age group.

Age Group	Number of Service Users
18–29	80
30–39	91
40–49	51
50–59	45
60–69	22

**Table 3:** Number of service users within each employment sector.

Employment Sector	Number of Service Users
Education	37
Emergency Services	9
Health Care (NHS)	106
Justice Services	6
Retail	18
Social Care	94
Supply and Logistics	10
Transport	9

Of consenting service users, 216 had logged on to the LLTTF Time for You Option 1 at least once. A large proportion (73.6%) of these service users logged on once only (N = 159); 27 (12.5%) logged on twice; 17 (7.9%) logged on three times; and a further 12 logged on between four and the maximum of 14 times. As all service users needed to log in to the LLTTF platform as part of registration, and therefore a large number of the single logins might have been from service users who self-referred to Options 2 and 3 and had, therefore, identified that Option 1 did not meet their needs. A total of 1,611 minutes were logged with a median (range 0 to 165) of 3 minutes per service user. Seventy-three service users accessed 258 modules and/or eBooks.

### Implementation facilitators

#### Adaptability to service user needs

It was reported that Option 2 and Option 3 offerings could be adapted to meet service users’ changing needs, possibly a greater adaptation from the planned guided CBT-focused service in Option 2. With low numbers accessing the service at times, it was possible for the Option 2 delivery team to be flexible with the length and number of sessions, offering more than the planned 20–30 minutes per week. An example of an adaptation at Option 2 was the introduction of additional signposting.

Trainee psychologists in Option 3 applied different therapeutic modalities to meet service user needs in line with intended delivery. Service users perceived the flexibility in times and number of sessions positively:

“Early on we established specific themes that we might want to go into in a bit more depth. I think as the weeks have gone on, you know, as I’ve been talking about something she’s maybe been able to be like, “you know it sounds like maybe something like this might be helpful.” So, we’ve kind of been able to look at some of the original intentions, but you know something like a therapist, I don’t think it was necessarily something that was raised initially, but it became really tied into some of the other stuff that I was talking about, so then that’s kind of maybe what led us to using some of those resources.”

(Option 2 Service User 030)

“We’ve not been given any boundaries in terms of how long a session should be. It could be up to two hours, but it could just be a check in email, you know, like it’s a really wide spectrum. And I think on the one hand that’s good, the fact that we can be flexible to respond to what somebody needs or what we think is helpful for them is it’s good to have that flexibility.”

(Option 2 Team Member 006)

#### Relatability of delivery staff to service users

Many of the service users worked in health and social care settings and some recognised and appreciated the similarity of their own role to that of the Wellbeing Practitioners and trainee psychologists. Option 2 had more of a peer-support quality than might have been expected, as service users recognised the similarities of the Wellbeing Practitioner role to the support they provided to colleagues in their own roles. In this way, Option 2 reflected more of a partnership approach between delivery staff and service users:

“I feel this is more of a partnership thing, working almost alongside them to help them, so they go off on their journey and you go off and help someone else. You’re almost sitting alongside them on their journey and it’s almost like a journey of discovery as well.” (Development Team Member 005)

#### Rapid access to support

An important facilitator of effectiveness was how quickly support could be accessed. There was immediate access to the Option 1 LLTTF resources, and contact within 48 hours for those choosing Option 2 and 3 for an intake assessment interview with a supportive element. In the early stages, the support practitioners contacted service users within 24–48 hours, although in later stages, there was around a 4-week wait for accessing the Option 3 service due to the increased demand on the service. Nonetheless, service users still perceived the offer as being very rapid compared with other services: “It was so quick, I remember being so shocked because in the past when I’ve, like I remember I was on that waiting list for ages.” (Option 3 Service User 028)

### Implementation barriers

#### Leadership disconnection between intervention development and delivery

Time for You was conceived as three distinct tiers of support, ranging from self-guided access to LLTTF, through guided access via short support sessions at Option 2, to a therapy-based intervention at Option 3. Service users would select their preferred option, with an intake assessment for Options 2 and 3 to ascertain the suitability of the preferred option based on level of client risk. The lack of a consistent project ‘champion’ through the development and delivery stages of the project led to disconnection between what was planned and implemented, particularly in relation to the purpose and format of the intake assessment. This disconnect led to some initial confusion and referrals of higher risk service users to trainee psychologists, with negative impacts on both the trainees and service users:

“I was just like so out of my depth that I was actually, this is going to cause more harm to her, like she’s telling me she doesn’t want short term therapy, she wants long term, but it had come through. And she was also telling me how she’d been let down by everybody, she was very much society’s let her down and I was like “well I’m going to let you down too, because I can’t do anything with you.”

(Trainee Psychologist 010)

“I’ve been very demoralised. And left with no support again.”

(Option 3 Service User 022)

An implementation plan might have enabled a stronger focus on standardised procedures specific to Time for You, supporting a closer fit with the planned service. Instead, initial processes, such as risk management plans, tended to be ‘borrowed’ from other services supporting individuals with more severe mental health challenges. These borrowed processes may have been less suited to the needs of frontline workers who were experiencing mild-to-moderate impacts on wellbeing but who were functioning well enough to self-refer to the Time for You service. The service recruited frontline delivery staff before the project management team were fully in place, which may explain this borrowing of processes that may not have been appropriate for the service users’ needs.

#### Lack of LLTTF engagement beyond first visit

A high number of single-session uses of LLTTF suggests a potential barrier to deeper engagement with the platform. SAMH staff had mixed feelings about whether LLTTF resources were easy to navigate, potentially due to their own preferences for face-to-face or more intensive support, as there was little negative feedback from service users who then came to Option 2. Wellbeing Practitioners reported service users who had previously found LLTTF resources helpful when they were using them independently, but who needed some support to stay engaged when their mood was low. However, service users reported finding access to LLTTF straightforward and talked of being able to find resources relatively easily.

These two views suggest issues with non-engagement at Option 1 may have been due to a mixture of needing initial guidance and perhaps occasional check-ins to retain motivation, similar to the original Option 2 plan: “…in terms of commitment and motivation, it’s helpful to have someone to check in with them and discuss it.” (WBP Option 2 Team Member 006)

### Acceptability and impact on service users

The support available through the different options available in the Time for You service seemed to be understood by, and were generally acceptable to, service users. The range of support suited different service user needs and goals. Adaptability was demonstrated within the Time for You service at all levels. In Option 1, the self-guided route, being able to access the support at times that suited service users was ideal for those on shift work and service users welcomed being able to select modules and other tools as they chose. In Options 2 and 3, some flexibility regarding meeting times, length of and attendance at sessions, and changing goals all contributed to acceptability.

“I know that some places they’re quite punitive, if you don’t attend appointments, or you don’t turn up, and they’re quite structured and I quite understand that its public service and resources are limited. And if you’re offering something for free, you want to add value to it. But there was not a punitive thing of you know, this is your final warning and if it happen. It kind of adapted to something that came up in my life unexpectedly. It wasn’t like, oh, well, this wasn’t part of what we were looking at. It wasn’t part of the structure. It was, okay, how can we support you round about that and kind of talk about feelings, which I really struggled with.” (Option 2 Service User 020)

#### Onwards referral pathways for more complex presentations

The Time for You service was not designed for service users with more severe and enduring mental health issues, but when these were identified either in the intake assessment interview, or later in the Option 2 or 3 support process service, users were signposted to alternative care pathways. The high demand for mental health support and long waiting lists were factors that were outside the Time for You service’s control but resulted in significant time and resource being used to support onward referral. Further consideration to how to meet this gap would be useful:

“There are people that have more complex needs… So, then what we do is look at maybe what we can put them on towards, whether that’s signposting, our onward referral path is fairly limited, we can’t refer into statutory services, but we can certainly signpost to their GP in the first instance if they do need that kind of clinical intervention.”

(Option 2 Team Member 012)

#### Impact on wellbeing

Service users had mixed success accessing and making use of LLTTF resources. The flexibility offered in time and use of the more relevant resources were valued, with one client comparing LLTTF to a “*troubleshooting guide*”, allowing the person to pick and choose what was needed to address a particular issue. Interviews with service users who engaged with the modules found them accessible and useful, and were able to discuss the content of specific modules and the benefit they had found from them:

“One that does ‘Face it, Fix it, Forget it’, about a worry. You know ’cause sometimes I get worries through your clients, you worry about them and stuff, but you know when you switch your phone off at 5:00 o’clock at night, you’re best to not think about your work. It’s easier said than done sometimes, eh. This is telling you to try to break your problem into chunks and trying to do it one piece at a time.”

(Option 1 Service User 15)

Those who had made use of resources reported a positive impact on their wellbeing. In some cases, engaging with LLTTF had given the service user the impetus to look for further support, by giving them a goal that they could work on:

“I think it’s actually been enough on its own now. Like at the start, it was still a struggle, but now that like I’ve finished it, I feel a lot better actually.” (Option 1, Service User 26);

“…when I was in a crisis point, it did help… but it was more because it changed the thought process. Now that was only a small change and it allowed a different conversation to happen, and I think actually it allows you when you’re going over and your brains working, you have something else to rely on to think, “oh yeah, yeah, ah yeah, I understand that”, but it took a bit of time.”

(Option 1, Service User 18)

SAMH Wellbeing Practitioners supported service users in Option 2, and those service users valued the flexibility of the service in adapting to their needs, especially as needs could change during the process:

“I started when I was in a really good headspace for starting and had the time and the head space for, I suppose, the website and looking at different things… the first few sessions were really, really helpful and chatted a bit more things than I realised would come up for it… my headspace altered from being in quite a clear place of I’ve got loads of time on my hands to do this work, to life been quite chaotic, and it has been supportive, talking about things.”

(Option 2, Service User 20)

Service users reported that having clear goals helped to maintain their focus and encouraged ongoing engagement with resources: “I’ve got people who’ve been so focused, and they know exactly the kind of the goals that they want to achieve, and that’s really helped to outline support sessions especially if they have the resources.” (Option 2, Service User 14).

Service users also reported that the support helped them re-engage with previous activities and to be able to recognise the contribution they were making and which they had lost sight of. It also increased confidence and self-compassion.

“Partly because of the support that I’ve had, I’ve now been able to maybe act on some of the issues that I’ve had. So, you know for example work related stress, like it’s been something that I’ve been building up to for a while, it’s kind of talking to my work about some of the issues that I have been having. So that would kind of have relied on things like having the compassion for myself to raise those issues and having the kind of confidence and kind of communication skills to bring things up in a way that’s effective.”

TfYC2030

The majority of Option 3 service users interviewed highly valued their contact with the trainee psychologists. One particularly valued aspect was the support that helped service users to understand how they were feeling and why, creating connections between aspects of their lives which were affecting their mood: “I don’t think I would have been able to make a lot of the connections that I have in my mind, without that support and without that guidance and that knowledge.” (Option 3, Client 16)

Service users also described how the support helped them to put down the issue causing them anxiety, instead of holding it in their head and going over it repeatedly. It contributed to service users feeling less judgemental about themselves and gaining more confidence in talking about how they were feeling:

“If I’m feeling low, it’s like I can just say it. Like I do that, I just feel if I do say to certain people like how I’m feeling, it maybe like not gets dismissed, but maybe just not understood properly. But at least it’s kind of giving me the confidence to kinda just say rather than just kind of bottling up inside and then it just kind of progresses and then gets worse.”

(Option 3, Client 17)

## Discussion

This process evaluation sought to consider an innovative service designed to support the mental health of frontline workers during the COVID-19 pandemic. A rapidly developed and implemented intervention, the Time for You service, provided service users with options including self-guided online CBT resources, guided engagement with these online CBT resources, or 1–1 psychological therapy with trainee sport and exercise and health psychologists. The intervention and its evaluation were implemented via a collaboration between a third sector mental health service (SAMH) and a university provider (GCU). Those service users who took part in interviews found the service to be an acceptable intervention. The dynamic combination of academic and third sector service development expertise made rapid implementation possible as trainees could be mobilised to deliver option 3 of the service quickly, combined with a highly responsive and client-focused delivery team. A more consistent link between the service development and delivery teams would have reduced drawbacks and ensured the development of the service aligned better with original plans.

### Intervention reach

The intervention evidenced excellent reach, with frontline workers from 31 of the 32 local authority areas in Scotland taking part. Service users also represented a range of different employment sectors ranging from education, emergency services, and justice services to health care, social care, and both retail and transport.

### Intervention facilitators

Services users appreciated the flexibility in appointment times and number of sessions offered by trainee psychologists in Option 3. Flexibility has been noted elsewhere as a benefit in the context of service provision relating to poor mental health and mental illness [e.g., 46–48]. However, should future service user numbers rise then the flexibility that trainee psychologists were able to offer may be reduced in order to, for example, manage caseloads where services are extended beyond the anticipated 6–8 sessions.

Service users also appreciated how quickly support could be accessed and this was an important facilitator of perceived effectiveness and satisfaction. Wait times ranged from immediate access to the Option 1 LLTTF resources through to an approximate 4-week wait for accessing the Option 3 service. Even in the context of a 4-week wait, service users still saw the service as one which was rapid and more responsive than other services. These findings reflect those reported in other healthcare settings where the time spent on waiting lists can be a strong predictor of subsequent satisfaction [49, 50] which is itself associated with better service user outcomes [51]. As such, the speed with which service users could access care was an important advantage for the service.

### Intervention barriers

Neither trainee health psychologists nor trainee sport and exercise psychologists are trained as part of British Psychological Society-accredited pathways to support service users with severe and enduring mental health issues who may already be accessing support from more intensive services. However, sometimes trainees on this service were put in a position where they were referred service users whom they did not have the professional competencies to support. This was a difficult situation for both trainees and service users. The key reason underpinning this challenge seemed to be a lack of continuity in staffing and leadership from the planning to the implementation stages of the intervention, partly explained by its rapid deployment within the context of the COVID-19 pandemic. It is recommended that a consistent project leader is present across all stages of any similar future intervention and that clear and explicit triaging is employed using appropriate, validated tools.

There was mixed evidence in relation to client engagement with the LLTTF platform. There were relatively high numbers of single-session users which reflected previous evaluations of LLTTF and similar services [32]. This may indicate a difficulty in using the platform, yet service users who had used it prior to progressing to Option 2 were positive about it. In addition, feasibility and acceptability evaluations of the platform have been positive [52] and service users themselves reported that it was easy to both use and to navigate. Trainees noted that some service users experiencing low mood may benefit from support to remain engaged with the material and it is possible that this may explain some of the infrequent use. In addition, as all service users who engaged with the service needed to initially log in to the LLTTF platform, the high number of single-session users may reflect a significant number of service users referred to Option 2 and Option 3 support not engaging with the platform beyond registration. That being said, a significant number of service users accessing the LLTTF platform did not engage beyond their first session suggesting they did not value the platform or it did not appear to meet their needs. More empirical data may be required in future to unpack competing explanations for the observed low-frequency interactions.

### Intervention acceptability and impact

The involvement of trainee psychologists delivering Option 3 of the service was acceptable to most service users and service users suggested that this support was equivalent to their other experiences of therapy. Evidence reviews have both supported the contention that trainees can effectively improve outcomes and rejected the notion that this is because trainees are allocated service users with less severe symptoms [39, 53].

The intervention was reported to be impactful for the interviewed service users in a number of different ways. The flexibility of the intervention was viewed as a strength in attaining impact as users felt they could tailor their use to what they most required. Additionally, service users reported that advances made during their engagement both encouraged a search for other support and helped them to maintain their engagement with the Time for You intervention itself. Therapeutic benefits reported by service users included helping them to better understand their feelings, feel less judgemental about themselves, and being more confident discussing their feelings with others.

Finally, there is a clear need to have defined, onward referral pathways for service users who present with problems that are beyond the scope of the support being offered. Such signposting was present in the Time for You intervention, but the experiences of trainees and service users suggested that it was sub-optimal. Effective triage systems may improve the client journey [54] though the availability of validated and effective ways of achieving this in non-urgent healthcare settings has been highlighted as a currently unresolved task [55] and may be particularly challenging when treatment options involve a mixture of both independent and supported treatment option as is the case with Time for You.

The evidence-base concerning the possible mechanisms explaining the impact of internet-based or face-to-face psychotherapies, including CBT, is inconsistent but does provide some suggestions for factors which may have influenced this service’s outcomes [56]. Terides et al. [57] found that increased CBT skills gained from self-guided internet-based CBT predicted improvement in symptoms and a higher satisfaction with life. Andersson et al. [56] also suggested that more user-friendly, relatively simple technology, along with a clear timeline for the length of treatment resulted in higher levels of effectiveness. More suggestions for time spent and regularity of engagement with the self-guided Option 1 offer might have therefore resulted in longer and more consistent engagement from services users.

### Strengths and limitations

A strength of this evaluation is the use of the Consolidated Framework for Implementation Research (CFIR) [41] to guide the work. In addition, the qualitative evaluation sampled and recruited service users who had either engaged with the service, not engaged beyond registration, or for whom the Time for You service had not been helpful. This approach provided an overview of mechanisms of positive or negative impacts in Options 1 and 3, although there were fewer service users from Option 2 in the qualitative sample. However, there were also limitations. First, we were unable to access useable data to quantitatively evaluate the impact of the intervention on service user mental health. The service intended using the WEMWBS measure as part of the assessment process and ongoing tracking of service user mental health, and our hope had been to access and use this data. Completion rates for the measure were poor and we therefore decided not to integrate the data within our evaluation as there was insufficient data to make any meaningful inferences about changes in mental health. Second, this evaluation did not involve stakeholders with lived experience (e.g., frontline workers experiencing mental health challenges) in the design or implementation of this evaluation. Such an approach can help inform insightful research questions as well as supporting an informed evaluation of data that are collected [58].

## Conclusions

Time for You was a rapidly developed and implemented intervention designed to support the mental health of frontline workers during the COVID-19 pandemic. In deploying a flexible online approach, the service allowed frontline workers to gain the support they needed, when they could engage with it, and in ways that supported their needs. Engaging trainee psychologists to deliver the online 1–1 therapeutic element of the service provided a flexible resource which reduced the waiting time for access to mental health services, and service users particularly appreciated this. The rapid implementation of the service did lead to some challenges in screening service users and low completion rates for the WEMWBS outcome measure, and future services should take steps to ensure that the scope and requirements of the service are clearly defined to service users and delivery teams.

## Supporting information

S1 File Interview schedules. This is the file containing example interview schedules.
(DOCX)

## References

[1] Bonati M, Campi R, Segre G. Psychological impact of the quarantine during the COVID-19 pandemic on the general European adult population: A systematic review of the evidence. Epidemiol Psychiatr Sci. 2022;31:e27. doi: 10.1017/S2045796022000051 35475479 PMC9069583

[2] Dubé JP, Smith MM, Sherry SB, Hewitt PL, Stewart SH. Suicide behaviors during the COVID-19 pandemic: A meta-analysis of 54 studies. Psychiatry Res. 2021 Jul 1;301:113998. doi: 10.1016/j.psychres.2021.113998 34022657 PMC9225823

[3] Richter D, Riedel-Heller S, Zürcher SJ. Mental health problems in the general population during and after the first lockdown phase due to the SARS-Cov-2 pandemic: rapid review of multi-wave studies. Epidemiol Psychiatr Sci. 2021;30:e27. doi: 10.1017/S2045796021000160 33685551 PMC7985862

[4] McPherson KE, McAloney-Kocaman K, McGlinchey E, Faeth P, Armour C. Longitudinal analysis of the UK COVID-19 Psychological Wellbeing Study: Trajectories of anxiety, depression and COVID-19-related stress symptomology. Psychiatry Res. 2021 Oct 1;304:114138. doi: 10.1016/j.psychres.2021.114138 34388511 PMC8424320

[5] O’Connor RC, Wetherall K, Cleare S, McClelland H, Melson AJ, Niedzwiedz CL, et al. Mental health and well-being during the COVID-19 pandemic: longitudinal analyses of adults in the UK COVID-19 Mental Health & Wellbeing study. Br J Psychiatry. 2021 Jun;218(6):326–33. doi: 10.1192/bjp.2020.212 33081860 PMC7684009

[6] Godefroy R, Lewis J. What explains the socioeconomic status-health gradient? Evidence from workplace COVID-19 infections. SSM Popul Health. 2022 Jun 1;18:101124. doi: 10.1016/j.ssmph.2022.101124 35669891 PMC9150908

[7] Khajuria A, Tomaszewski W, Liu Z, Chen JH, Mehdian R, Fleming S, et al. Workplace factors associated with mental health of healthcare workers during the COVID-19 pandemic: an international cross-sectional study. BMC Health Serv Res. 2021 Dec;21:1–1. doi: 10.1186/s12913-021-06279-6 33743674 PMC7981382

[8] Schmuck J, Hiebel N, Rabe M, Schneider J, Erim Y, Morawa E, et al. Sense of coherence, social support and religiosity as resources for medical personnel during the COVID-19 pandemic: A web-based survey among 4324 health care workers within the German Network University Medicine. PLOS ONE. 2021;16(7). doi: 10.1371/journal.pone.0255211 34310616 PMC8312980

[9] Clarissa C, Quinn S, Stenhouse R. ‘Fix the issues at the coalface and mental wellbeing will be improved’: a framework analysis of frontline NHS staff experiences and use of health and wellbeing resources in a Scottish health board area during the COVID-19 pandemic. BMC Health Serv Res. 2021 Dec;21:1–1. doi: 10.1186/s12913-021-07103-x 34645439 PMC8513559

[10] Lebel C, MacKinnon A, Bagshawe M, Tomfohr-Madsen L, Giesbrecht G. Elevated depression and anxiety symptoms among pregnant individuals during the COVID-19 pandemic. J Affect Disord. 2020 Dec 1;277:5–13. doi: 10.1016/j.jad.2020.07.126 32777604 PMC7395614

[11] Wang K, Manning RB III, Bogart KR, Adler JM, Nario-Redmond MR, Ostrove JM, et al. Predicting depression and anxiety among adults with disabilities during the COVID-19 pandemic. Rehabil Psychol. 2022 Jan 27. doi: 10.1037/rep0000434 35084914

[12] Lizana PA, Lera L. Depression, anxiety, and stress among teachers during the second COVID-19 wave. Int J Environ Res Public Health. 2022 May 14;19(10):5968. doi: 10.3390/ijerph19105968 35627505 PMC9140393

[13] Houghton S, Kyron M, Hunter SC, Lawrence D, Hattie J, Carroll A, et al. Adolescents’ longitudinal trajectories of mental health and loneliness: The impact of COVID‐19 school closures. J Adolesc. 2022 Feb;94(2):191–205. doi: 10.1002/jad.12017 35353417 PMC9087620

[14] Kibbey MM, Fedorenko EJ, Farris SG. Anxiety, depression, and health anxiety in undergraduate students living in initial US outbreak “hotspot” during COVID-19 pandemic. Cogn Behav Ther. 2021 Sep 3;50(5):409–21. doi: 10.1080/16506073.2020.1853805 33433271

[15] Sánchez-García JC, Cortés-Martín J, Rodríguez-Blanque R, Marín-Jiménez AE, Montiel-Troya M, Díaz-Rodríguez L. Depression and anxiety in patients with rare diseases during the COVID-19 pandemic. Int J Environ Res Public Health. 2021 Mar 21;18(6):3234. doi: 10.3390/ijerph18063234 33800980 PMC8003983

[16] Dozois DJ. Anxiety and depression in Canada during the COVID-19 pandemic: A national survey. Canadian Psychology / Psychologie canadienne. 2021;62(1):136–42. doi: 10.1037/cap0000251

[17] Shevlin M, McBride O, Murphy J, Miller JG, Hartman TK, Levita L, et al. Anxiety, depression, traumatic stress and COVID-19-related anxiety in the UK general population during the COVID-19 pandemic. BJPsych Open. 2020 Nov;6(6):e125. doi: 10.1192/bjo.2020.109 33070797 PMC7573460

[18] Wu S, Zhang K, Parks-Stamm EJ, Hu Z, Ji Y, Cui X. Increases in anxiety and depression during COVID-19: a large longitudinal study from China. Front Psychol. 2021 Jul 6;12:706601. doi: 10.3389/fpsyg.2021.706601 34295294 PMC8290070

[19] Th’ng F, Rao KA, Ge L, Mao D, Neo HN, Molina JA, et al. A one-year longitudinal study: changes in depression and anxiety in frontline emergency department healthcare workers in the COVID-19 pandemic. Int J Environ Res Public Health. 2021 Jan;18(21):11228. doi: 10.3390/ijerph182111228 34769750 PMC8583330

[20] Firat M, Okanli A, Kanbay Y, Utkan M, Demir Gokmen B. The prevalence of pandemic anxiety, anxiety and depression during the COVID-19 pandemic in Turkey. Psychiatry and Clinical Psychopharmacology. 2021;31(2):198–205. doi: 10.5152/pcp.2021.21641 PMC11079677 38765224

[21] Kujawa A, Green H, Compas BE, Dickey L, Pegg S. Exposure to COVID‐19 pandemic stress: Associations with depression and anxiety in emerging adults in the United States. Depress Anxiety. 2020 Dec;37(12):1280–8. doi: 10.1002/da.23109 33169481 PMC13347965

[22] Lakhan R, Agrawal A, Sharma M. Prevalence of depression, anxiety, and stress during COVID-19 pandemic. J Neurosci Rural Pract. 2020 Oct;11(04):519–25. doi: 10.1055/s-0040-1716442 33144785 PMC7595780

[23] Greene T, Harju-Seppänen J, Adeniji M, Steel C, Grey N, Brewin CR, et al. Predictors and rates of PTSD, depression and anxiety in UK frontline health and social care workers during COVID-19. Eur J Psychotraumatol. 2021 Jan 1;12(1):1882781. doi: 10.1080/20008198.2021.1882781 33968317 PMC8075082

[24] Mirimoghaddam MM, Ahmadi R, Sezavar M, Ahmadi A, Soleimani Houni M, Hosseinnataj A, et al. Comparing Anxiety and Depression among Midwives and Nurses Working in Pediatric Wards and Other Clinical Settings during the Covid-19 Outbreak. Int J Pediatr. 2022 Jun 1;10(6):16195–204. doi: 10.22038/ijp.2021.57880.4541

[25] Quintana-Domeque C, Lee I, Zhang A, Proto E, Battisti M, Ho A. Anxiety and depression among medical doctors in Catalonia, Italy, and the UK during the COVID-19 pandemic. PLoS One. 2021 Nov 2;16(11):e0259213. doi: 10.1371/journal.pone.0259213 34727110 PMC8562811

[26] Roberts NJ, McAloney-Kocaman K, Lippiett K, Ray E, Welch L, Kelly C. Levels of resilience, anxiety and depression in nurses working in respiratory clinical areas during the COVID pandemic. Respir Med. 2021 Jan 1;176:106219. doi: 10.1016/j.rmed.2020.106219 33248362 PMC7648185

[27] Sumner RC, Kinsella EL. Grace under pressure: Resilience, burnout, and wellbeing in frontline workers in the United Kingdom and Republic of Ireland during the SARS-CoV-2 pandemic. Front Psychol. 2021 Jan 27;11:576229. doi: 10.3389/fpsyg.2020.576229 33584412 PMC7874970

[28] Weibelzahl S, Reiter J, Duden G. Depression and anxiety in healthcare professionals during the COVID-19 pandemic. Epidemiol Infect. 2021;149:e46. doi: 10.1017/S0950268821000303 33557984 PMC7900665

[29] Cogan N, Kennedy C, Beck Z, McInnes L, MacIntyre G, Morton L, et al. ENACT study: What has helped health and social care workers maintain their mental well‐being during the COVID‐19 pandemic?. Health Soc Care Community. 2022 Nov;30(6):e6656–73. doi: 10.1111/hsc.13992 36068667 PMC9539329

[30] Pappa S, Athanasiou N, Sakkas N, Patrinos S, Sakka E, Barmparessou Z, et al. From recession to depression? Prevalence and correlates of depression, anxiety, traumatic stress and burnout in healthcare workers during the COVID-19 pandemic in Greece: A multi-center, cross-sectional study. Int J Environ Res Public Health. 2021 Mar 1;18(5):2390. doi: 10.3390/ijerph18052390 33804505 PMC7967750

[31] Bowyer HL, Pegler R, Williams C. Computerized cognitive behaviour therapy for anxiety and depression in farming communities: mied methods feasibility study of participant use and acceptability. JMIR Form Res 2023; 7. doi: 10.2196/42573 37335597 PMC10337352

[32] Elison S, Ward J, Williams C, Espie C, Davies G, Dugdale S, et al. Feasibility of a UK community-based, eTherapy mental health service in Greater Manchester: repeated-measures and between-groups study of ‘Living Life to the Full Interactive’,‘Sleepio’and ‘Breaking Free Online’at ‘Self Help Services’. BMJ Open. 2017 Jul 1;7(7):e016392. doi: 10.1136/bmjopen-2017-016392 28729322 PMC5541623

[33] Pittaway S, Cupitt C, Palmer D, Arowobusoye N, Milne R, Holttum S, et al. Comparative, clinical feasibility study of three tools for delivery of cognitive behavioural therapy for mild to moderate depression and anxiety provided on a self‐help basis. Ment Health Fam Med. 2009 Sep;6(3):145. 22477905 PMC2838647

[34] ) National Institute for Health and Care Excellence (2022). Depression in adults: treatment and management. [Nice Guideline NG222]. https://www.nice.org.uk/guidance/ng222 35977056

[35] Andrews G, Basu A, Cuijpers P, Craske MG, McEvoy P, English CL, et al. Computer therapy for the anxiety and depression disorders is effective, acceptable and practical health care: an updated meta-analysis. J Anxiety Disord. 2018 Apr 1;55:70–8. doi: 10.1016/j.janxdis.2018.01.001 29422409

[36] Carlbring P, Andersson G, Cuijpers P, Riper H, Hedman-Lagerlöf E. Internet-based vs. face-to-face cognitive behavior therapy for psychiatric and somatic disorders: an updated systematic review and meta-analysis. Cogn Behav Ther. 2018 Jan 2;47(1):1–8. doi: 10.1080/16506073.2017.1401115 29215315

[37] Iqbal Z, Airey ND, Brown SR, Wright NM, Miklova D, Nielsen V, et al. Waiting list eradication in secondary care psychology: Addressing a National Health Service blind spot. Clin Psychol Psychother. 2021 Jul;28(4):969–77. doi: 10.1002/cpp.2551 33415754

[38] Cardno SJ, Sahraie A. The expanding backlog of mental health patients: Time for a major rethink in COVID-19 policy. 2020, April; doi: 10.31234/osf.io/st5b2

[39] Nyman SJ, Nafziger MA, Smith TB. Client outcomes across counselor training level within a multitiered supervision model. J Couns Dev. 2010 Apr;88(2):204–9. doi: 10.1002/j.1556-6678.2010.tb00010.x

[40] Moore GF, Audrey S, Barker M, Bond L, Bonell C, Hardeman W, et al. Process evaluation of complex interventions: Medical Research Council guidance. Brit Med J. 2015 Mar 19;350. doi: 10.1136/bmj.h1258 25791983 PMC4366184

[41] Damschroder LJ, Aron DC, Keith RE, Kirsh SR, Alexander JA, Lowery JC. Fostering implementation of health services research findings into practice: a consolidated framework for advancing implementation science. Implement Sci. 2009 Dec;4(1):1–5. doi: 10.1186/1748-5908-4-50 19664226 PMC2736161

[42] Keith RE, Crosson JC, O’Malley AS, Cromp D, Taylor EF. Using the Consolidated Framework for Implementation Research (CFIR) to produce actionable findings: a rapid-cycle evaluation approach to improving implementation. Implement Sci. 2017 Dec;12(1):1–2. doi: 10.1186/s13012-017-0550-7 28187747 PMC5303301

[43] ) Scottish Association for Mental Health. *86% of frontline workers burnt out by the pandemic*. https://www.samh.org.uk/about-us/news-and-blogs/86-of-frontline-workers-in-scotland-burnt-out-by-the-pandemic [Accessed 22nd June 2023]

[44] Tennant R, Hiller L, Fishwick R, Platt S, Joseph S, Weich S, et al. The Warwick-Edinburgh mental well-being scale (WEMWBS): development and UK validation. Health Qual Life Outcomes. 2007 Dec;5(1):1–3. doi: 10.1186/1477-7525-5-63 18042300 PMC2222612

[45] Clarke V, Braun V, Hayfield N. Thematic analysis. Qualitative psychology: A practical guide to research methods. 2015 Jan 1;3:222–48.

[46] Lester H, Marshall M, Jones P, Fowler D, Amos T, Khan N, et al. Views of young people in early intervention services for first-episode psychosis in England. Psychiatr Servi. 2011 Aug;62(8):882–7. doi: 10.1176/ps.62.8.pss6208_0882 21807826

[47] Lund K, Hultqvist J, Bejerholm U, Argentzell E, Eklund M. Group leader and participant perceptions of Balancing Everyday Life, a group-based lifestyle intervention for mental health service users. Scand J Occup Ther. 2020 Aug 17;27(6):462–73. doi: 10.1080/11038128.2018.1551419 30706746

[48] O’Toole MS, Ohlsen RI, Taylor TM, Purvis R, Walters J, Pilowsky LS. Treating first episode psychosis–the service users’ perspective: a focus group evaluation. J Psychiatr Ment Health Nurs. 2004 Jun;11(3):319–26. doi: 10.1111/j.1365-2850.2004.00730.x 15149380

[49] Marsden J, Stewart D, Gossop M, Rolfe A, Bacchus L, Griffiths P, et al. Assessing client satisfaction with treatment for substance use problems and the development of the Treatment Perceptions Questionnaire (TPQ). Addiction Research. 2000 Jan 1;8(5):455–70. doi: 10.3109/16066350009005590

[50] Xie Z, Or C. Associations between waiting times, service times, and patient satisfaction in an endocrinology outpatient department: a time study and questionnaire survey. Inquiry. 2017 Nov 21;54:0046958017739527. doi: 10.1177/0046958017739527 29161947 PMC5798665

[51] Glickman SW, Boulding W, Manary M, Staelin R, Roe MT, Wolosin RJ, et al. Patient satisfaction and its relationship with clinical quality and inpatient mortality in acute myocardial infarction. Circ Cardiovasc Qual Outcomes. 2010 Mar;3(2):188–95. doi: 10.1161/CIRCOUTCOMES.109.900597 20179265

[52] Williams C, McClay CA, Martinez R, Morrison J, Haig C, Jones R, et al. Online Cognitive Behavioral Therapy (CBT) Life Skills Program for Depression: Pilot Randomized Controlled Trial. JMIR Form Res. 2022 Feb 17;6(2):e30489. doi: 10.2196/30489 35175203 PMC8895278

[53] Vos J, Chryssafidou E, van Rijn B, Stiles WB. Outcomes of beginning trainee therapists in an outpatient community clinic. Couns Psychother Res. 2022 Jun;22(2):471–9. doi: 10.1002/capr.12466

[54] Harding KE, Taylor NF, Leggat SG. Do triage systems in healthcare improve patient flow? A systematic review of the literature. Aust Health Rev. 2011 Aug 25;35(3):371–83. doi: 10.1071/AH10927 21871201

[55] Déry J, Ruiz A, Routhier F, Bélanger V, Côté A, Ait-Kadi D, et al. A systematic review of patient prioritization tools in non-emergency healthcare services. Syst Rev. 2020 Dec;9:1–4. doi: 10.1186/s13643-020-01482-8 33023666 PMC7541289

[56] Andersson G, Titov N, Dear BF, Rozental A, Carlbring P. Internet‐delivered psychological treatments: from innovation to implementation. World Psychiatry. 2019 Feb;18(1):20–8. doi: 10.1002/wps.20610 30600624 PMC6313242

[57] Terides MD, Dear BF, Fogliati VJ, Gandy M, Karin E, Jones MP, et al. Increased skills usage statistically mediates symptom reduction in self-guided internet-delivered cognitive–behavioural therapy for depression and anxiety: a randomised controlled trial. Cogn Behav Ther. 2018 Jan 2;47(1):43–61. doi: 10.1080/16506073.2017.1347195 28724338

[58] Beresford P. User involvement, research and health inequalities: developing new directions. Health Soc Care Community. 2007 Jul;15(4):306–12. doi: 10.1111/j.1365-2524.2007.00688.x 17578391

