# Supplementary material for: Time for You: A process evaluation of the rapid implementation of a multi-level mental health support intervention for frontline workers during the COVID-19 pandemic
Source: PLoS One. 2023 Oct 27;18(10):e0293393. doi: 10.1371/journal.pone.0293393 (PMC10610521; doi:10.1371/journal.pone.0293393)
Supplement: S1 File — This is the file containing example interview schedules. (DOCX) [file pone.0293393.s001.docx]

**S1 File**

**Interview Schedule – Frontline Workers**

Each of the following questions/topics will be covered during the semi-structured interview with participants, although possibly not in the following order or using the exact terms provided below. Interviewers will follow up content of interest, ask for examples to illustrate content, and prompt participants for detail throughout the interviews.

1. What is/was the nature of your frontline role during the COVID-19 pandemic?
2. What have been/were your experiences of working on the frontline during the COVID-19 pandemic?
3. What were the reasons for you seeking support from the Time for You service?
4. Which tier of the Time for You project were you involved in?
5. Please describe your experience of gaining support through the Time for You project?
6. How has your involvement in the Time for You project helped you address some of the challenges that prompted you to seek support?
7. How do you feel the Time for You project and service be improved?

---

**Interview Schedule – GCU Trainee Psychologists**

Each of the following questions/topics will be covered during the semi-structured interview with participants, although possibly not in the following order or using the exact terms provided below. Interviewers will follow up content of interest, ask for examples to illustrate content, and prompt participants for detail throughout the interviews.

1. What is/was the nature of your role within the Time for You project?
2. Can you please describe how you supported Frontline Workers accessing the Time for You project at Tier 3 of the project?
3. How would you describe the overall implementation of the Time for You project in relation to the initial plan for the project? Please describe any changes to the planned project delivery, and provide context as to why these changes occurred and the outcomes of these changes.
4. How would you describe participant engagement with Tier 3 of the Time for You project?
5. How would you describe the influence of the Time for You project on the mental health and wellbeing of the frontline workers the project supported?
6. How did you feel providing support to frontline workers as part of the Time for You project?
7. How would you describe the experience of providing support in a more general way to support the mental health and wellbeing of frontline workers rather than those clients you are normally used to supporting (i.e., in counselling, health and/or sport & exercise contexts)?
8. How do you feel the Time for You project and service be improved?

---

**Interview Schedule – SAMH Wellbeing Practitioners**

Each of the following questions/topics will be covered during the semi-structured interview with participants, although possibly not in the following order or using the exact terms provided below. Interviewers will follow up content of interest, ask for examples to illustrate content, and prompt participants for detail throughout the interviews.

1. What is/was the nature of your role within the Time for You project?
2. Can you please describe how you supported Frontline Workers accessing the Time for You project for each Tier of Support?
3. Can you please describe your experience of delivering support as part of the Time for You project?
4. Can you please describe how frontline workers were allocated to tiers of support?
5. How would you describe the overall implementation of the Time for You project in relation to the initial plan for the project? Please describe any changes to the planned project delivery, and provide context as to why these changes occurred and the outcomes of these changes.
6. How would you describe participant engagement with each of the tiers of the Time for You project?
7. How would you describe the influence of the Time for You project on the mental health and wellbeing of the frontline workers the project supported?
8. How do you feel the Time for You project and service be improved?

---

**Interview Schedule – Project Stakeholders**

Each of the following questions/topics will be covered during the semi-structured interview with participants, although possibly not in the following order or using the exact terms provided below. Interviewers will follow up content of interest, ask for examples to illustrate content, and prompt participants for detail throughout the interviews.

1. What is/was the nature of your role in relation to the Time for You project?
2. Can you please describe how the Time for You project was developed? What perceived need let to the project’s development? What informed the approach taken within the Time for You project?
3. How would you describe the overall implementation of the Time for You project in relation to the initial plan for the project? Please describe any changes to the planned project delivery, and provide context as to why these changes occurred and the outcomes of these changes.
4. How would you describe participant engagement with the Time for You project across each tier of the service?
5. How would you describe the influence of the Time for You project on the mental health and wellbeing of the frontline workers the project supported?
6. How do you feel the Time for You project and service be improved?
